# Chloroform Condemned

**Published:** 1875-03

**Authors:** J. Hardman

**Affiliations:** Muscatine, Iowa


### CHLOROFORM CONDEMNED.

BY J. HARDMAN, D. D. S., MUSCATINE, IOWA.

The question of the moral justification in the use of chloroform for general anæsthesia, in dental and surgical practice, has often had its claims forcibly impressed upon us. Should we, as a profession, having the entire control of the patients placing themselves unreservedly in our care for treatment, use so deadly and so treacherous an agent as chloroform has proven itself to be? For over twenty years since, we have ourself refused to use this agent for anæsthesia. Our convictions were, and remain the same, that no security against its fatal tendency is known; no reliable rules by which to select a subject exempt from its deadly influence; no antidotes efficient, or means of resuscitation deserving of much confidence.

We took occasion in a paper we had the honor to read before the Iowa State Dental Society in 1867, (and which was published in the DENTAL REGISTER of Jan. 1868) to state somewhat in detail our views in regard to this subject.

It is gratifying to see that the profession in the State of Massachusetts are moving in the right direction, as is indicated by the following clipping from the Press:

"The Massachusetts Dental Society has passed resolutions emphatically condemning the use of chloroform as an anæsthetic, and declaring any member administering it liable to expulsion."

This has the correct ring, and shows morality coupled with enterprise.

At the last meeting of the Iowa State Dental Society, we presented the following preambles and resolutions:

"*Whereas*, the frequency of death caused by the use of chloroform as an anæsthetic, is justly exciting apprehension and alarm in the people; and

"*Whereas*, after protracted years in its use, no security against its deadly tendency, either in the mode of its administration or selection of subject has been developed; and

"*Whereas*, no truly respectable profession should ignore the value of moral rectitude in practice, should with impunity hazard the lives of its patrons, or heedlessly disregard the intelligent warnings of statistical record; therefore

"RESOLVED, That we the members of the Iowa State Dental Society, condemn the use of chloroform for the purpose of general anæsthesia, and will refrain from so using it in our practice. And, moreover, we will use our influence to favor its entire expulsion as an anæsthetic agent."

The discussion of this resolution elicited the too prevailing fact, that many dental practitioners go forward without pausing to reflect as to the moral responsibility that should and must accompany the conduct of the dentist apart from mere expedience. The comparative exemption from legal accountability, in connection with its very general popularity, are cited as evidence that no great amount of crime can attend its use. But more frequent penalties such as was obtained by the estate of J. W. Lawrence, of Floyd County, Iowa, against Dr. C. C. Birney, in which the jury found a verdict of $1,750 for malpractice, in using chloroform on the deceased to a fatal termination, will probably be the only means of arousing a just apprehension of this subject.

The above resolution was laid upon the table, and will again be called up, when, it is hoped, that our Society will place its condemnation upon this sad practice by an unanimous vote, establishing by so doing its well merited position of dignified honor.

We have no especial object at this time save to put in the minds of the friends of true principle, correct conduct and dignified humanity in our profession to concentrate their efforts and influence to the breaking up of this dangerous practice in the use of chloroform

We trust that at the dental conventions which may meet for deliberation during the present year, all may take a decided action in this matter, to the end that the showing will be emphatically in favor of abandoning its further use.

